# Parental dietary patterns and assisted reproductive technology outcomes including embryo morphokinetics: rotterdam periconception cohort

**DOI:** 10.1007/s10815-025-03688-y

**Published:** 2025-10-25

**Authors:** Batoul Hojeij, Sam Schoenmakers, Lenie van Rossem, Sten Willemsen, Esther Baart, Melek Rousian, Régine P. M. Steegers-Theunissen

**Affiliations:** 1 Department of Obstetrics and Gynaecology, Erasmus MC, University Medical Center, Rotterdam, 3015GD The Netherlands; 2 Division of Human Nutrition and Health, Wageningen University and Research, Wageningen, 6708 PB The Netherlands; 3 Division of Reproductive Endocrinology and Infertility, Department of Obstetrics and Gynaecology, Erasmus MC, University Medical Center, Rotterdam, 3015GD The Netherlands; 4 Department of Developmental Biology, Erasmus MC, University Medical Center, Rotterdam, 3015GD The Netherlands

**Keywords:** Diet, Embryo development, Time-lapse imaging, Fertility, Assisted reproductive technology

## Abstract

**Purpose:**

This exploratory study investigated the associations between dietary patterns of subfertile couples and assisted reproductive technology (ART) outcomes, including preimplantation embryo morphokinetics.

**Methods:**

From the ongoing Rotterdam periconception cohort, we included 149 women and 126 men attending a fertility outpatient clinic for ART treatment. Dietary intake was assessed using a validated self-reported food frequency questionnaire with implausible dietary reporters excluded. We identified four dietary patterns in women and men separately. Embryo morphokinetics included the timing of each division from two to eight cell stage (t2 to t8) and to blastocyst formation, second and third cell cycle and synchrony, and the Known Implantation Data score on embryonic day 3 (KIDscore D3). ART outcomes included the fertilization rate, embryo yield, clinical pregnancy, and live birth.

**Results:**

Maternal adherence to the “Healthy” pattern was associated with shorter S2 (β_adj_ −0.62 h, *p* = 0.024) and higher KIDscore D3 (OR_adj_ 0.78, *p* = 0.011), while “Savory Snack and Alcohol” pattern was associated with slower t6 (β_adj_ 0.869 h, *p* = 0.014), t7 (β_adj_ 1.63 h, *p* < 0.001), and t8 (β_adj_ 1.52 h, *p* = 0.01). Paternal adherence to the “Healthy” pattern was associated with faster t7 (β_adj_ −1.10 h, *p* = 0.046), t8 (β_adj_ −2.12 h, *p* = 0.001), and shorter S3 (β_adj_ −1.72 h, *p* = 0.001), while “Potato-rich” pattern was associated with faster t2 (β_adj_ −0.46 h, *p* = 0.012). Parental dietary patterns were not associated with ART outcomes.

**Conclusions:**

This study showed small but consistent associations between parental diet and preimplantation embryo morphokinetics, with no overall effects on ART outcomes.

**Supplementary Information:**

The online version contains supplementary material available at 10.1007/s10815-025-03688-y.

## Introduction

Subfertility is an increasing public health problem affecting around 17.5% of couples worldwide [1]. Female factor subfertility accounts for 50%, while male factor subfertility contributes to 20–30%, and 20–30% is attributed to a combination of both female and male factors [2]. Despite advances in assisted reproductive technology (ART) treatments and technological improvement (e.g., time-lapse imaging), the accumulative live birth rates after ART are plateauing, with potential deleterious consequences on the psychological, social, and financial levels of subfertile couples [3–6]. Evidence from the last two decades shows that maternal and paternal lifestyle behaviors have a direct impact on fertility, preimplantation embryo development and ART outcomes [7–10].

Assessment and selection of the most optimal embryos for transfer is a fundamental step for ART treatment success. Conventional methods are primarily based on morphological grading of embryos, based upon one time-point [11]. The introduction of time-lapse imaging enables continuous and precise monitoring of embryonic dynamics and development [12, 13]. Embryo morphokinetics have been associated with ART outcomes including implantation, pregnancy, and live birth rates [14–16]. Furthermore, this technology allows for investigating associations between various parental exposures and embryo morphokinetics at critical stages, which can help identify factors that influence early embryo development and subsequent ART outcomes. The Known Implantation Data (KID) score is a tool based on time-lapse imaging, developed to assist in selecting embryos during ART treatment, regardless of fertilization method and culture medium [17, 18]. When applied to day 3 embryos, the scoring system can predict the implantation potential of embryos, and has been shown to be more effective than conventional methods in predicting live births [17, 19]. Among the parental lifestyle behaviours, accumulating evidence from animal and human studies demonstrate a profound effect of diet on preimplantation embryo development and ART outcomes [7–9, 20, 21]. Diet influences parental gametes, which are key determinants of preimplantation embryo development including the morphokinetics [22–25]. The underlying mechanisms involve inflammation modulation, redox regulation, and providing micronutrients relevant for gametogenesis [22, 26, 27]. Subsequently, these effects can have long-lasting implications on reproductive outcomes including pregnancy and live birth [15, 16, 28, 29]. For example, nutrients such as folate, antioxidants, and omega-3 fatty acids improve oocyte and sperm quality, which directly contributes to preimplantation embryo morphokinetics [23, 30–34]. Furthermore, intake of food groups such as whole grains, meat and fish, and micronutrients such as folate, antioxidant vitamins, as well as omega-3 fatty acids have been associated with ART outcomes [9, 35–37]. Literature on the mechanisms by which preimplantation embryo morphokinetics influence the ART outcomes is limited; however, there is some evidence that these embryonic features reflect aspects of embryo competence. Embryo morphokinetics provide information on genetic competence and segregation, with optimal kinetics being associated with euploidy and proper segregation [16, 38]. It also reflects aspects of epigenetic and transcriptomic profiles, where disruptions in these processes may manifest as suboptimal kinetics (i.e., very fast or slow) [39, 40]. Furthermore, morphokinetic parameters can serve as indicators of blastocyst quality which contributes to the chance of pregnancy and live birth [41, 42]. Thus, understanding the association between parental diet and embryo morphokinetics can help identify modifiable dietary behaviours that optimize ART outcomes and provide evidence-based dietary guidance for subfertile couples.

Instead of focusing on isolated dietary components, analysis of dietary patterns allows for capturing the complex interactions and synergistic effects between nutrients and foods, providing a comprehensive assessment of diet as a whole [43, 44]. Dietary patterns are commonly derived using a priori and a posteriori methods [45]. A priori approaches rely on predefined diet quality indices based on existing knowledge such as Mediterranean diet and Healthy Eating indices, and reflect adherence to healthy or unhealthy dietary behaviours [43, 45]. On the other hand, *posteriori* methods such as principal component analysis (PCA) and cluster analysis are data-driven approaches that identify naturally occurring dietary patterns based on combinations of foods consumed in the population [43, 45].

Studies to date mainly investigated the impact of maternal dietary patterns on ART outcomes [10]. On the other hand, data on the association between parental dietary patterns and preimplantation embryo morphokinetics is extremely limited [7, 46, 47]. One study in couples undergoing ART treatment assessed the effect of a Mediterranean dietary intervention and showed accelerated embryo morphokinetics and improved KIDscore day 3 [47]. This exploratory study aims to investigate associations between maternal and paternal dietary patterns separately and preimplantation embryo morphokinetics, and ART outcomes, focusing on fertilization rate, embryo yield, and clinical pregnancy and live birth rates. Secondary outcomes include associations between maternal and paternal dietary patterns and, respectively, the number of retrieved and metaphase II (MII) oocytes and total motile sperm count (TMSC).

## Material and methods

### Study design and population

Between May 2017 and December 2021, 763 women and 679 men were enrolled in the Virtual Embryoscope study, a subcohort embedded in the Rotterdam Periconception Cohort (the Predict study) [48, 49].

**Fig. 1:**
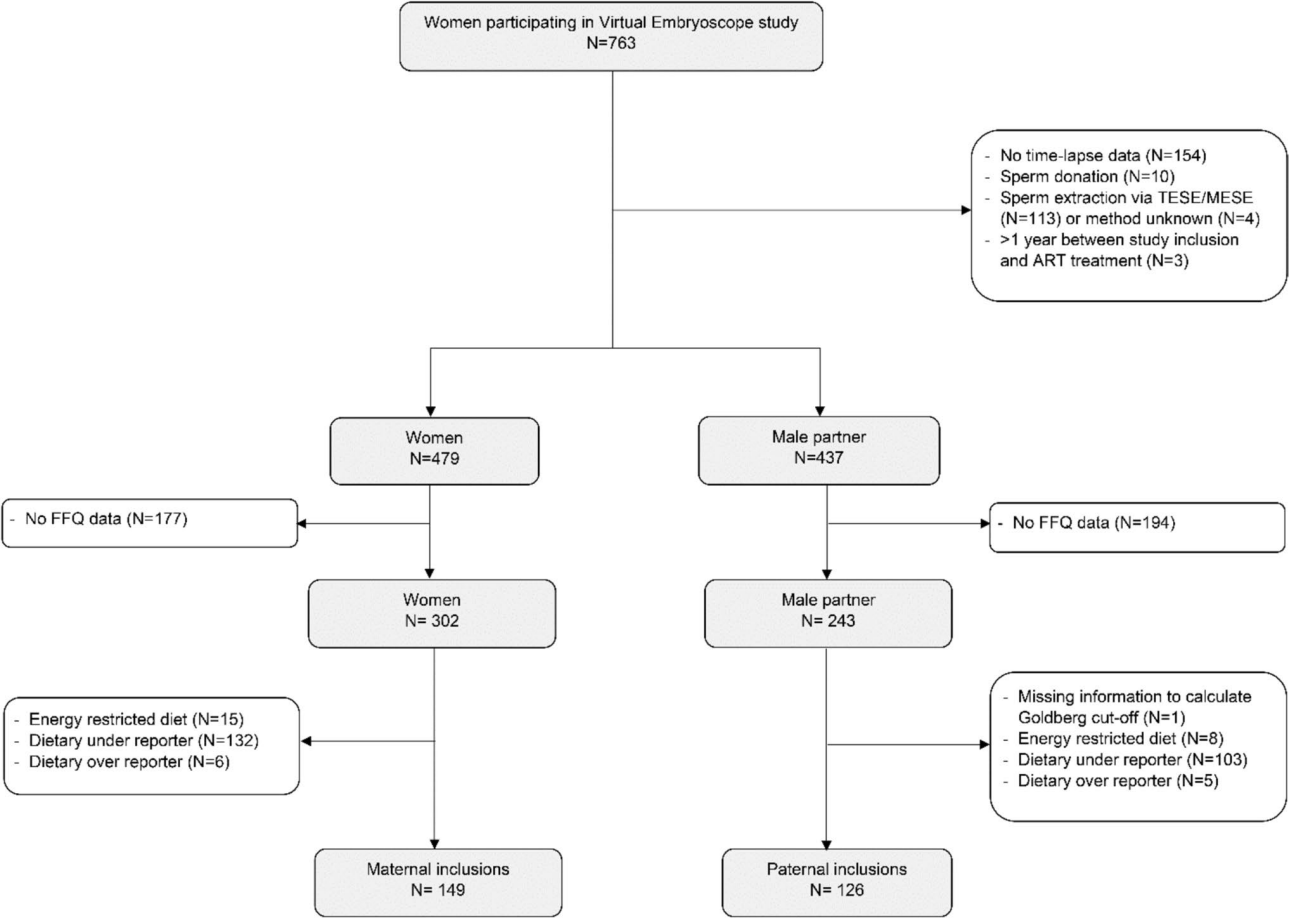
Flowchart of study population. *ART* assisted reproductive technology, *FFQ* food frequency questionnaire, *TESE/MESE* testicular/microsurgical epididymal sperm extraction

Included participants were planned to undergo ART treatment (in vitro fertilization (IVF) with or without intracytoplasmic sperm injection (ICSI)), of at least 18 years of age and had a good command of the Dutch language. Participants were excluded if no time-lapse data from embryo or data from food frequency questionnaires (FFQ) were available, sperm was extracted by means of testicular or microsurgical epididymal sperm extraction methods, or unknown sperm extraction method, or more than 1 year between inclusion and ART treatment (Fig. 1). In addition, participants were excluded if they were on an energy-restricted diet, or were identified as dietary under- and over-reporters by use of the Goldberg method (Fig. 1) [50]. The upper and lower Goldberg cut-off limits were calculated at the individual level as described by Black (Supplementary information 1) [50].

### Assessment of dietary intake

Preconceptional diet was assessed with a self-administered 190-item FFQ. The FFQ was developed by the division of Human Nutrition, Wageningen University, the Netherlands based on data from the Dutch National Food Consumption Survey and validated for energy, macronutrients and B vitamins intake [51, 52]. Participants were asked to recall the frequency of consumption (no consumption up to 7 days per week) and portion size consumed (e.g., in tablespoons, cups, slices, pieces) over the past month. Based on the frequency of intake and portion size, the daily intake of the 190 food items was calculated (grams/day) as well as the total energy intake (calories). To identify the dietary patterns, food items were grouped into 26 predefined food groups based on similarities in origin and nutritional composition [43, 53].

### *In vitro* fertilization treatment procedures

Ovarian stimulation was performed using recombinant follicle stimulating hormone (rFSH) or urinary FSH co-treated with gonadotropin-releasing hormone (GnRH) agonist or antagonist according to standard protocols of the Erasmus MC [54, 55]. Follicle maturation was triggered using human chorionic gonadotrophin (hCG) or GnRH agonist. Thereafter, oocytes were retrieved and transferred to culture medium (SAGE human tubal fluid with 5% human serum albumin, Cooper Surgical, Trumbull, CT, USA or G-IVF plus, Vitrolife, Goteborg, Sweden).

ART procedures were performed according to standard protocols as described previously [54, 55]. Prior to IVF or ICSI treatment, semen was analyzed according to World Health Organization (WHO) criteria to determine ejaculate volume (WHO 2010), concentration (WHO 2010) and motility (WHO 1999) [56, 57]. The TMSC was calculated by multiplying the volume, with concentration, and percentage of fast (sperm grade A) plus slow (sperm grade B) progressive sperms, divided by 100. Men with TMSC of ≤ 1 × 10^6^ were considered primarily for ICSI treatment. For ICSI treatment, motile sperm were manually selected, and injected into mature oocytes at the MII stage.

### Embryo culture and grading

Fertilized oocytes were transferred for culturing in a time-lapse incubator (EmbryoScope, Vitrolife Goteborg, Sweden) in EmbryoSlides (Vitrolife) filled with either SAGE-1 step Cooper Surgical (Trumbull, CT, USA) or G-TL plus (Vitrolife), after pronuclei inspection for IVF treatment and oocyte injection for ICSI treatment [58, 59]. Embryos were cultured at 37 °C, 7% oxygen and 5% (SAGE1-step) or 6% (G-TL plus) carbon dioxide.

During culture in the Embryoscope®, embryos were graded based on morphological criteria using a single image obtained at day 3 (May 2017 to April 2019) or day 5 (April 2019 to December 2021) [54]. From April 2019, embryo selection for transfer was extended locally to day 5 due to changes in laboratory policy and procedures. Time-lapse parameters were not used for embryo selection. Morphological criteria used for grading and selection of embryos for transfer on day 3 were the following: blastomere number and equality in size of cells, fragmentation and signs of early compaction. A top quality embryo consisted of eight blastomeres which are compact with < 10% fragmentation and a difference in cell size. Day 5 embryos were evaluated according to the Gardner embryo grading system, which describes the stage of blastocyst expansion on a numerical scale from 1 to 6 and qualities of the trophectoderm and inner cell mass using grades 1–3. Top quality blastocysts had an expansion score of 4 or higher and an inner cell mass or trophectoderm grade of 1.

### Time-lapse imaging and assessment of embryo morphokinetics

**Fig. 2:**
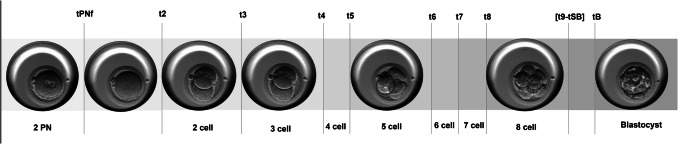
Embryo time-lapse parameters. *PN* pronuclei, *tPNf* timing to PN fading, *t2 to t8* timing to two- until eight-cell stages, *t9-tSB* timing to nine-cell stage until start of blastulation, *tB* timing to blastocyst formation

Embryo images were recorded automatically in seven focal planes every 10–15 min. Annotations of morphokinetic information were performed manually for transferred and cryopreserved embryos by trained members of our department using the EmbryoViewer® software. The intraclass correlation coefficient for inter-observer reproducibility for annotations was > 0.95 from pronuclei until t5 cell stage and 0.23–0.40 for t-6, 7- and 8-cell stages [60]. Definitions of time-points evaluated in this study are as follows: tPNf, timing to pronuclear fading; t2 to t8, timing to reach the 2-, 3-, 4-, 5-, 6-, 7-, and 8-cell stage; and tB, timing to blastocyst formation (Fig. 2). We defined tPNf as t0, to account for the time difference between IVF and ICSI for the moment of fertilization [61]. Additionally, we calculated the second and third cell cycle duration (CC2 = t3-t2; CC3 = t5-t3, respectively) and time of synchrony (S2 = t4-t3; S3 = t8-t5, respectively), as they have previously been described to be indicative of embryo quality [62].

A KIDscore was automatically generated for each embryo by the EmbryoViewer® software based on the KIDscore Day 3 (D3) and Day 5 (D5) scoring systems [17, 18]. The KIDscore D3 (ordinal score) can be applied for day 3 and day 5 embryos, whereas KIDscore D5 (continuous score) can only be applied to day 5 embryos. To have a comparable and harmonized outcome for all embryos, we retrospectively calculated the KIDscore D3 for day 3 and day 5 freshly transferred and cryopreserved embryos. The KIDscore D3 ranges from 1 to 5 and uses five time-lapse parameters: score 1, t3-tPNf (cut-off value = 11.48 h); score 2, t3 (cut-off value = 42.91 h); score 3, (t5-t3)/(t5-52) (cut-off value = 0.3408 h); score 4, (t5-t3)/(t5-52) (cut-off value = 0.5781 h); score 5, t8 (cut-off value = 66.0 h). Internal validation of the KIDscore D3 at our clinical department showed that embryos with a KIDscore D3 of 1 implant in 23% of cases, and KIDscore D3 of 5 implant in 52% of cases [60].

### Assessment of ART treatment outcomes

ART outcomes were obtained from electronic medical records. Fertilization rate was defined as the number of fertilized oocytes with two pronuclei divided by the number of retrieved oocytes for IVF cycles, and MII oocytes for ICSI cycles (Supplementary information 2). Embryo yield was defined as the number of usable embryos (transferred or frozen) divided by the number of fertilized oocytes with two pronuclei (Supplementary information 2). Clinical pregnancy was confirmed by an ultrasound for the presence of a heartbeat (early pregnancy) or for a viable fetus (mid to late pregnancy). Live birth rate was defined as live birth per the cycle of embryo transfer.

### Statistical analysis

Continuous baseline data are presented as medians with interquartile ranges and categorical baseline data are presented as absolute numbers with percentages.

PCA was used to derive naturally occurring dietary patterns in the study population, with varimax rotation of the 26 food groups. The PCA was conducted separately for the maternal and paternal data to ensure that the derived patterns were independent of the partner’s diet. Four dietary patterns were selected based on scree plot and Eigenvalue > 1.0. The factor loadings were calculated for each food group which represent contribution of food group(s) to a particular dietary pattern. Dietary patterns were labeled according to food groups with factor loading ≥ 0.4. Factor scores for each of the four dietary patterns were calculated for each participant by summing the intake of food groups weighted by the factor loadings. Higher factor scores represent higher adherence to a particular dietary pattern.

Linear mixed models were used to analyze the associations between selected dietary patterns and embryo morphokinetics. The KIDscore D3 was modelled using a continuation ratio model including a random intercept for each model to account for clustering. The continuation ratio model can be considered as a discrete version of the more well-known survival models for time-to-event outcomes where the ordinal response (here the KIDscore D3) assumes the role of the time variable [63]. In the (forward) continuation ratio model, we estimated the odds for an embryo to remain at a certain KIDscore D3 level rather than being above that level. Thus, when the odds ratio is < 1, the expected KIDscore D3 is higher and vice versa. Fertilization rate and embryo yield were analyzed using linear regression models. Clinical pregnancy and live birth rates were analyzed using firth logistic regression models. All models were adjusted for maternal and paternal age, BMI, geographic origin (Western/Non-Western), daily calorie intake, smoking status (Yes/No), alcohol consumption (Yes/No), folic acid and/or dietary supplement use (Yes/No) and conception mode (IVF/ICSI). When analyzing clinical pregnancy rate, the model was additionally adjusted for day of embryo transfer. Maternal dietary supplement use was not included in the model to analyze live birth rate since all women used folic acid as supplement.

Outcomes on embryo morphokinetics, pregnancy and live birth rates were analyzed using R software (version 4.1.3); fertilization rate, embryo yield and oocyte and TMSC outcomes were analyzed using SPSS software (version 28.0.1.0). *P* ≤ 0.05 was considered statistically significant.

## Results

### Baseline characteristics

Maternal and paternal baseline characteristics are summarized in Table 1.  A total of 149 women and 126 men were included in the final analysis of this study. The median maternal and paternal ages were 34 and 35 years, respectively. Maternal and paternal BMI were in the normal range, with median values of 22.7 kg/m² and 24.7 kg/m², respectively. The majority of women were of Western geographic origin (N=127, 85.2%), used folic acid and/or dietary supplement (N=140, 94.0%), and consumed alcohol (N=82, 55.0%), and were less likely to smoke (N=18, 12.1%). The majority of men were of Western geographic origin (N=107, 84.9%), consumed alcohol (N=83, 65.9%), and were less likely to smoke (N=22, 17.5%) or to use dietary supplements (N=44, 34.9%).

**Table 1:** Maternal and paternal baseline characteristics

Characteristics	Maternal (*N* = 149)	Paternal (*N* = 126)
Age (years), median (IQR)	34.34 (31.11–37.53)	34.84 (31.78–38.65)
BMI (Kg/m^2^), median (IQR)	22.70 (20.76–25.18)	24.72 (22.88–27.30)
Geographic origin, N (%)		
Western	127 (85.20)	107 (84.90)
Non-Western	15 (10.10)	11 (8.70)
Missing, *N* (%)	7 (4.70)	8 (6.30)
Education level, N (%)		
Low	2 (1.30)	5 (4.00)
Moderate	45 (30.20)	44 (34.90)
High	96 (64.50)	69 (54.80)
Missing, *N* (%)	6 (4.00)	8 (6.30)
Smoker, *N* (%)	18 (12.10)	22 (17.50)
Missing, *N* (%)	7 (4.70)	9 (7.10)
Alcohol consumption, *N* (%)	82 (55.00)	83 (65.90)
Missing, *N* (%)	7 (4.70)	9 (7.10)
Dietary supplement use, *N* (%)	140 (94.00)	44 (34.90)
Missing, *N* (%)	7 (4.70)	8 (6.30)
Insemination method		
IVF	66 (44.30)	60 (47.60)
ICSI	83 (55.70)	66 (52.40)
Embryo transfer		
No embryo transfer, *N* (%)	18 (12.08)	13 (10.32)
Day 3, *N* (%)	82 (55.03)	71 (56.35)
Day 5, *N* (%)	44 (29.53)	34 (26.98)
Missing, *N* (%)	5 (3.36)	8 (6.35)

*ICSI* Intracytoplasmic sperm injection, *IVF* in vitro fertilization, *IQR* interquartile range.

### Maternal and paternal dietary patterns

Four major dietary patterns were identified by PCA among women and men, which explained 32.9% and 34.3% of the total variance, respectively (Supplementary Table 1). The maternal dietary patterns were labeled “Healthy,” “Potato and Meat,” “Eggs, Legumes and Fruit and Vegetable juices” and “Savory Snack and Alcohol” and they explained 10.5%, 8.6%, 7.2%, and 6.7% of variance, respectively. The paternal dietary patterns were labeled “Healthy,” “Egg and Meat,” “Potato-rich,” and “Snack, Alcohol and Coffee,” and they explained 9.1%, 9.0%, 8.4%, and 7.8% of variance, respectively.

### Maternal and paternal dietary patterns in relation to preimplantation embryo morphokinetics

Associations between maternal and paternal dietary patterns with preimplantation embryo morphokinetics and KIDscore D3 are shown in Table 2 and Table 3, respectively. The average number of embryos assessed per participant was 4 (SD ± 3). Higher maternal adherence to the “Healthy” dietary pattern was associated with shorter S2 (β_adj_ −0.62 h; 95%CI −1.16, −0.08; *p* = 0.02), while the “Savory Snack and Alcohol” dietary pattern was associated with slower development to the six- (β_adj_ 0.87 h; 95%CI 0.18, 1.56; *p* = 0.01), seven- (β_adj_ 1.63 h; 95%CI 0.81, 2.46; *p* < 0.001) and eight-cell stages (β_adj_ 1.51 h; 95%CI 0.35, 2.68; *p* = 0.01). No associations were observed between maternal adherence to the two other dietary patterns and any of the embryo morphokinetics. Higher maternal adherence to “Healthy” dietary pattern was also associated with higher chance of improved KIDscore D3 (i.e., a lower chance the embryo remains at the same KIDscore D3 level, than to progress to a higher level) (OR_adj_ 0.78; 95%CI 0.63, 0.96; *p* = 0.01). No associations were observed between the other three maternal dietary patterns with KIDscore D3.

**Table 2:** Associations between maternal dietary patterns and embryo morphokinetics and KIDscore D3

Maternal dietary patterns									
		**Healthy**		**Potato and meat**		**Eggs, legumes and fruit and vegetable juices**		**Savory snack and alcohol**	
	***N* (N**_**embryo**_**)**	***β* (95%CI)**	***P*-value**	***β* (95%CI)**	***P*-value**	***β* (95%CI)**	***P*-value**	***β* (95%CI)**	***P*-value**
t2-tPNf	147 (559)	−0.186 (**−**0.382, 0.010)	0.062	0.032 (**−**0.173, 0.237)	0.756	−0.182 (−0.403, 0.040)	0.107	0.175 (−0.020, 0.369)	0.077
t3-tPNf	145 (542)	0.460 (−0.089, 1.009)	0.099	**−**0.266 (**−**0.828, 0.297)	0.350	0.596 (−0.023, 1.214)	0.058	0.302 (−0.235, 0.839)	0.266
t4-tPNf	145 (539)	−0.053 (−0.628, 0.523)	0.855	**−**0.169 (**−**0.748, 0.409)	0.561	−0.006 (−0.654, 0.642)	0.985	0.473 (−0.070, 1.016)	0.086
t5-tPNf	143 (525)	0.598 (−0.250, 1.445)	0.164	**−**0.369 (**−**1.219, 0.482)	0.390	0.918 (−0.026, 1.861)	0.056	0.431 (−0.388, 1.249)	0.298
t6-tPNf	141 (510)	0.160 (−0.612, 0.932)	0.681	**−**0.614 (**−**1.366, 0.137)	0.107	0.708 (−0.126, 1.543)	0.094	**0.869 (0.178, 1.561)**	**0.014**
t7-tPNf	138 (497)	0.108 (−0.853, 1.070)	0.823	**−**0.178 (**−**1.138, 0.781)	0.712	0.234 (−0.818, 1.287)	0.658	**1.632 (0.809, 2.455)**	**0.0002**
t8-tPNf	132 (471)	−0.137 (−1.412, 1.137)	0.830	**−**0.118 (**−**1.422, 1.186)	0.857	0.618 (−0.775, 2.012)	0.378	**1.515 (0.354, 2.676)**	**0.011**
tB-tPNf^a^	60 (203)	0.508 (−3.316, 4.334)	0.783	**−**0.057 (**−**3.718, 3.604)	0.974	3.376 (−0.405, 7.157)	0.077	0.431 (−3.809, 4.670)	0.833
CC2	148 (554)	0.656 (−0.043, 1.354)	0.065	**−**0.261 (**−**0.970, 0.448)	0.466	0.517 (−0.264, 1.298)	0.191	−0.128 (−0.812, 0.555)	0.709
CC3	146 (537)	−0.027 (−0.644, 0.590)	0.930	**−**0.033 (**−**0.658, 0.591)	0.916	0.192 (−0.501, 0.885)	0.583	0.006 (−0.593, 0.604)	0.984
S2	148 (551)	**−0.619 (−1.155, −0.082)**	**0.024**	0.088 (**−**0.478, 0.654)	0.757	−0.599 (−1.211, 0.012)	0.054	0.152 (−0.387, 0.690)	0.576
S3	135 (482)	−0.402 (−1.651, 0.847)	0.523	0.590 (**−**0.686, 1.867)	0.359	−0.446 (−1.815, 0.924)	0.518	1.096 (−0.063, 2.255)	0.063
	**N**_**embryo**_	**OR (95%CI)**	***P*-value**	**OR (95%CI)**	***P*-value**	**OR (95%CI)**	***P*-value**	**OR (95%CI)**	***P*-value**
KID3	137 (480)	**0.780 (0.634, 0.961)**	**0.011**	0.891 (0.714, 1.111)	0.304	0.920 (0.713, 1.188)	0.523	1.185 (0.959, 1.463)	0.116

*β* Beta coefficient, *CI* confidence interval, *CC2* duration of second cell cycle, *CC3* duration of third cell cycle, *KID3* Known Implantation Data Day 3, *OR* odds ratio, *S2* second cell cycle synchrony, *S3* third cell cycle synchrony, *tB* timing to blastocyst formation, *tPNf* timing to pronuclei fade, *t2-t8* timing to form 2- to 8-cells division stages. Significant associations (p < 0.05) are presented in bold.

Model 1: Adjusted for maternal and paternal age, BMI, geographic origin, daily calorie intake, smoking, alcohol consumption, and dietary supplement use and conception mode.

^a^ Model 1 but no adjustment for maternal dietary supplement use.

**Table 3:** Associations between paternal dietary patterns and embryo morphokinetics and KIDscore D3

Paternal dietary patterns									
		**Healthy**		**Eggs and meat**		**Potato-rich**		**Snack, alcohol and coffee**	
	***N* (N**_**embryo**_**)**	***β* (95%CI)**	***P*-value**	***β* (95%CI)**	***P*-value**	***β* (95%CI)**	***P*-value**	***β* (95%CI)**	***P*-value**
t2-tPNf	125 (464)	−0.123 (−0.533, 0.286)	0.550	−0.036 (−0.420, 0.349)	0.497	**−0.461 (−0.818, −0.130)**	**0.012**	0.311 (−0.060, 0.681)	0.099
t3-tPNf	123 (448)	−0.084 (−0.816, 0.648)	0.820	−0.157 (−0.840, 0.527)	0.649	−0.631 (−1.282, 0.020)	0.057	0.072 (−0.600, 0.744)	0.832
t4-tPNf	123 (445)	0.048 (0.725, 0.821)	0.902	−0.419 (−1.136, 0.297)	0.248	−0.421 (−1.114, 0.272)	0.231	0.269 (−0.440, 0.978)	0.453
t5-tPNf	120 (434)	−0.135 (−1.119, 0.850)	0.786	−0.241 (−1.167, 0.685)	0.606	−0.428 (−1.329, 0.473)	0.347	0.276 (−0.639, 1.191)	0.550
t6-tPNf	116 (424)	−0.685 (−1.620, 0.251)	0.149	−0.228 (−1.112, 0.657)	0.609	−0.776 (−1.617, 0.065)	0.070	0.200 (−0.672, 1.071)	0.650
t7-tPNf	115 (411)	**−1.095 (−2.172, −0.018)**	**0.046**	−0.535 (−1.556, 0.486)	0.300	−0.656 (−1.647, 0.334)	0.191	−0.008 (−1.030, 1.014)	0.987
t8-tPNf	110 (383)	**−2.122 (−3.372, −0.872)**	**0.001**	−0.022 (−1.303, 1.259)	0.972	−0.808 (−2.026, 0.410)	0.190	−0.008 (−1.252, 1.236)	0.989
tB-tPNf^a^	50 (169)	−1.013 (−3.693, 1.666)	0.444	−1.512 (−6.874, 3.850)	0.567	0.585 (−1.491, 2.661)	0.568	−0.060 (−2.246, 2.127)	0.955
CC2	124 (457)	0.056 (−0.679, 0.790)	0.880	−0.003 (−0.683, 0.677)	0.993	−0.253 (−0.907, 0.402)	0.445	−0.276 (−0.939, 0.387)	0.410
CC3	121 (443)	−0.060 (−0.645, 0.525)	0.838	−0.095 (−0.666, 0.475)	0.741	0.083 (−0.474, 0.640)	0.768	0.135 (−0.441, 0.710)	0.642
S2	124 (454)	0.107 (−0.484, 0.699)	0.719	−0.242 (−0.802, 0.319)	0.394	0.168 (−0.381, 0.717)	0.544	0.186 (−0.374, 0.745)	0.511
S3	111 (390)	**−1.723 (−2.774, −0.672)**	**0.001**	0.042 (−1.056, 1.141)	0.939	−0.183 (−1.245, 0.878)	0.731	−0.475 (−1.537, 0.587)	0.375
	**N**_**embryo**_	**OR (95%CI)**	***P*-value**	**OR (95%CI)**	***P*-value**	**OR (95%CI)**	***P*-value**	**OR (95%CI)**	***P*-value**
KID3	112 (389)	0.870 (0.705, 1.073)	0.193	0.991 (0.810, 1.212)	0.929	0.943 (0.772, 1.151)	0.563	0.984 (0.803, 1.204)	0.873

* β* Beta coefficient, *CI* confidence interval, *CC2* duration of second cell cycle, *CC3* duration of third cell cycle, *KID3* Known Implantation Data Day 3, *OR* odds ratio, *S2* second cell cycle synchrony, *S3* third cell cycle synchrony, *tB* timing to blastocyst formation, *tPNf* timing to pronuclei fade, *t2-t8,* timing to form 2- to 8-cells division stages. Significant associations (p < 0.05) are presented in bold.

Model 1: Adjusted for maternal and paternal age, BMI, geographic origin, daily calorie intake, smoking, alcohol consumption, and dietary supplement use and conception mode.

^a^ Model 1 but no adjustment for maternal dietary supplement use.

Higher paternal adherence to the “Healthy” dietary pattern was associated with faster development to the seven- (β_adj_ −1.10 h; 95%CI −2.17, −0.02; *p* = 0.046) and -eight cell stages (β_adj_ −2.12; 95%CI −3.37, −0.87; *p* = 0.001), and shorter S3 (β_adj_ −1.72 h; 95%CI −2.77, −0.67; *p* = 0.001), and the “Potato-rich” dietary pattern was associated with faster development to two-cell stage (β_adj_ −0.46 h; 95%CI −0.82, −0.13; *p* = 0.012). Paternal adherence to the two other dietary patterns was not associated with any of the embryo morphokinetics, and no associations were found between any of the paternal dietary patterns with KIDscore D3.

### Maternal and paternal dietary patterns in relation to ART treatment outcomes

No significant associations were observed between any of maternal and paternal dietary patterns and ART outcomes (fertilization rate, embryo yield, clinical pregnancy and live birth). However, maternal adherence to the “Savory Snack and Alcohol” dietary pattern was associated with a 6% higher embryo yield (i.e., 6% more usable embryos relative to the number of fertilized oocytes with two pronuclei) (β_adj_ 6.36; 95%CI 0.63, 12.08; *p* = 0.03) (Table 4).

**Table 4:** Associations between maternal and paternal dietary patterns and ART treatment outcomes

	**Maternal dietary patterns**				**Paternal dietary patterns**			
	**Healthy**	**Potato and meat**	**Eggs, legumes and fruit and vegetable juices**	**Savory snack and alcohol**	**Healthy**	**Egg and meat**	**Potato- rich**	**Snack, alcohol and coffee**
Fertilization rate^a^								
β (95%CI)	−2.244 (−7.565, 3.076)	4.044 (−1.069, 9.157)	−0.259 (−5.989, 5.471)	−3.315 (−8.417, 1.787)	−1.014 (−3.360, 4.332)	2.351 (−2.578, 7.280)	−0.426 (−4.996, 4.145)	1.284 (−3.519, 6.088)
P-value	0.404	0.119	0.929	0.200	0.707	0.346	0.854	0.597
Embryo yield^a^								
β (95%CI)	1.498 (−4.605, 7.601)	−2.329 (−8.244, 3.586)	−3.998 (−10.491, 2.495)	**6.356** (**0.632, 12.079**)	−0.838 (−7.290, 5.614)	−0.443 (−6.422, 5.535)	−4.064 (−9.497, 1.368)	0.234 (−5.554, 6.021)
P-value	0.627	0.436	0.224	**0.030**	0.797	0.883	0.141	0.936
Clinical pregnancy^b^								
OR (95%CI)	1.157 (0.693, 2.028)	0.952 (0.561, 1.587)	1.190 (0.690, 2.062)	1.040 (0.651, 1.690)	1.098 (0.643, 1.891)	0.995 (0.517, 1.869)	0.778 (0.475, 1.241)	1.066 (0.671, 1.790)
P-value	0.581	0.850	0.526	0.868	0.731	0.983	0.291	0.790
Live birth^c^								
OR (95% CI)	1.275 (0.757, 2.147)	1.076 (0.629, 1.840)	1.259 (0.723, 2.190)	0.994 (0.623, 1.590)	1.207 (0.710, 2.051)	1.028 (0.681, 1.550)	0.779 (0.488, 1.243)	1.109 (0.692, 1.777)
P-value	0.360	0.788	0.414	0.983	0.487	0.897	0.295	0.665

*β* Beta coefficient, *CI,* confidence interval, *ART* assisted reproductive technology, *OR* odds ratio. N of men and women included in the analysis, respectively: fertilization rate, 144 and 124; embryo usage rate, 143 and 124; clinical pregnancy 130 and 111; live birth, 126 and 105. Significant associations (p < 0.05) are presented in bold.

^a^ Model 1: Adjusted for maternal and paternal age, BMI, geographic origin, daily calorie intake, smoking, alcohol consumption, and dietary supplement use and conception mode.

^b^ Model 1 and day of embryo transfer.

^c^ Model 1 but no adjustment for maternal dietary supplement use.

### Maternal and paternal dietary patterns in relation to fertility parameters

No associations were found between maternal and paternal dietary patterns and respectively oocyte or sperm quality (Supplementary Table 2).

## Discussion

In this study, maternal adherence to the “Healthy” dietary pattern was associated with faster preimplantation embryonic development and higher chance of improved KIDscore D3, whereas the “Savory Snack and Alcohol” dietary pattern was associated with slower preimplantation embryonic development. Paternal adherence to the “Healthy” dietary pattern was also associated with faster preimplantation embryonic development, and the “Potato-rich” dietary pattern was associated with faster development to the two cell stage. However, maternal and paternal dietary patterns were not associated with overall ART outcomes.

Potential maternal and paternal effects on preimplantation embryo development are mediated by oocyte and sperm quality, respectively [64, 65]. The positive effects of maternal and paternal healthy dietary pattern on preimplantation embryo morphokinetics effects can be attributed to the quantity of fruits, vegetables, nuts and seeds content of the pattern [7, 66, 67]. These foods are rich in antioxidant vitamins and omega-3 fatty acids, and have been shown to positively affect embryo quality [68–72]. Antioxidants protect against oxidative stress which can cause oocyte and sperm DNA damage [26, 27]. Oxidative stress can negatively impact preimplantation embryo development through several mechanisms including spindle and chromosomal abnormalities, irregular oocyte mitochondrial morphology and decreased mitochondrial mass, as well as sperm chromatin alterations and impairment of DNA demethylation [71, 73, 74]. By mitigating these effects, antioxidants promote optimal embryo development. Notably, antioxidants and omega-3 fatty acids support normal mitochondrial function [75]. Mitochondria plays a pivotal role in early embryo development through multiple processes such as producing Adenosine triphosphate (ATP) to meet energy demands during development, maintaining redox balance, and producing intermediate metabolites that support epigenetic regulation and gene expression [76]. Besides, the “Healthy” dietary pattern is also a good source of folate, which supports one-carbon metabolism [77]. Indeed, the one-carbon metabolism plays an important role in DNA synthesis and methylation [77]. Furthermore, vegetables and nuts have also shown an impact on sperm DNA methylation at specific regions [78, 79]. During preimplantation embryo development, the transmitted epigenetic profile undergoes a wave of DNA (de)methylation occurs, which is essential for regulating gene expression and guiding optimal embryonic development [80]. Thus, disruptions of the epigenetic profile in gametes can dysregulate gene expression, thereby impacting embryo development [81]. Comparable to our findings, Kermack et al. showed a positive association between parental Mediterranean diet and faster embryo morphokinetics and KIDscore D3, while Hoek et al. observed similar association between maternal vegetable intake and KIDscore D3 [7, 47].

Although the paternal healthy dietary patterns was associated with faster t7, t8, and S3, no significant association was observed with KIDscore Day 3. One possible explanation is that a number of time-points included in the score (e.g., t2, t3) were not influenced by the paternal healthy diet, preventing a significant change in the KIDscore. It could also be that maternal (epi)genetic factors have a stronger impact on embryo quality at early cleavage stages compared to paternal factors [82]. Moreover, the embryo relies on the oocyte mitochondria during early cleavage stages (e.g., t2, t3), which provides the energy required for cell division and thus can have greater influence on early embryo kinetics compared to sperm [83–85]. A previous study from our group showed a positive association between maternal vegetable intake and KIDScore day 3, whereas paternal vegetable intake showed no significant association [7].

The maternal “Savory Snack and Alcohol” dietary pattern represents an unhealthy and inadequate diet consisting of energy dense, high salt and nutrient-poor food, which could negatively impact oocyte quality, thus affecting early embryo development [21, 25, 30, 86–88]. For example, restriction of protein in the diet resulted in altered amino acid metabolism and abnormal mitochondria of oocytes and cumulus cells of rats [86]. High-salt diet also lead to altered spindle assembly and chromosomal alignment in mice [89, 90]. Moreover, the low antioxidant content of this pattern combined with the pro-oxidant effects of alcohol [70, 91, 92], can increase oxidative stress, potentially impacting oocyte and embryo quality as described previously [65, 71, 93]. Although not directly comparable, two previous studies showed a positive association between unhealthy lifestyle factors (smoking, overweight and obesity) and slower embryo morphokinetics [94, 95].

It is worth noting that, it is still unclear which intracellular processes are affected by dietary patterns, and thus why certain time-points are affected over others. Comparable to our findings, previous studies showed associations between lifestyle factors and specific time-lapse parameters over others [60, 94–96].

Our findings showed an unexpected, positive association between maternal “Savory Snack and Alcohol” dietary pattern and the proportion of usable embryos. The reason behind this association is unclear especially that the “Savory Snack and Alcohol” dietary pattern was associated with slower kinetics, suggesting compromised embryo development. Also, prenatal exposure to alcohol has detrimental effects on preimplantation embryo development [97]. One possible explanation relates to the standard clinical practices: in case of advanced maternal age, there may be a decision for double embryo transfer (86% of cases with double embryo transfers had a maternal age > 38 years); and in couples where only a single embryo is available, it will be transferred in almost all cases if it shows any signs of life (embryo yield was 100% when only one fertilized oocyte with two pronuclei was available). Thus, although it could mean a high yield would be obtained, it certainly does not indicate better quality. Furthermore, embryo yield is a rough estimation for embryo quality since all the embryos selected to be transferred or frozen are indicated as usable, without ranking each embryo’s morphological quality.

Previous studies demonstrated an improved clinical potential of faster cleaving embryos in terms of implantation potential, pregnancy chance, and live birth [14–16]. Despite healthy dietary pattern association with faster embryo morphokinetics in our study, we did not find an association between maternal and paternal dietary patterns and clinical pregnancy and live birth rates. One possible explanation is that embryos selected for transfer are based on morphological quality and the KIDscore D3, ignoring the embryonic morphokinetics. Also, it might be that the time-points that were associated with diet do not have great influence on pregnancy and live birth, or that the dietary effects on morphokinetics were insufficient to affect these outcomes. For example, t5 has been reported to be associated with implantation, as well as to be different between the pregnancy/live birth and non-pregnancy/no live birth groups [98–102]. In our study, however, none of the dietary patterns were associated with t5. Following conception, uterine factors (e.g., anatomic defects, endometrial thickness and receptivity) play an important role in implantation, pregnancy, and live birth [103, 104]. Uterine anatomical defects impede embryo implantation, while endometrial thickness and receptivity are important to support embryo attachment and growth [103, 104]. Hence, if uterine factors are suboptimal, they could attenuate the positive effects of the healthy diet on preimplantation embryo development. Also, the uterine environment involves processes such as hormonal signaling, immune activity, and inflammation, which can also influence the maintenance of pregnancy and live birth [105]. Besides diet, the uterine environment is further influenced by maternal characteristics and exposures such as genetics, age, smoking, stress, and infections [106]. In other words, the effect of diet on preimplantation embryo development and its translation to pregnancy and live birth outcomes is modulated by a variety of maternal factors that can attenuate the dietary effect. Besides, if diet-induced epigenetic modifications occurred, they may have either been on inherited genes that do not influence embryo developmental competence, or, if they did, they might have been reset during epigenetic reprogramming in early embryonic development [81]. Nevertheless, these are proposed mechanisms that should be tested in future research. In line with our observations, a previous study by Kermack et al. showed an association between the Mediterranean diet with embryo morphokinetics, but observed no effects on pregnancy and live birth [47]. Moreover, the majority of studies on associations between maternal and paternal dietary patterns and ART outcomes showed no associations, comparable to our findings [10, 107, 108]. In addition, a recent meta-analysis showed no association between maternal healthy dietary patterns, that had overlapping food items with the “Healthy” dietary pattern such as fruits, vegetable, and fish, with ART treatment success [36].

A key strength of this study is its prospective design with clinically relevant outcomes for ART treatment. Time-lapse imaging allowed for the study of temporal associations between parental dietary patterns and preimplantation embryo morphokinetics until blastocyst stage. In this study, dietary patterns were assessed for 149 women (61 with partner dietary data available and 88 without) and 126 men (61 with partner dietary data available and 65 without). Analyses of the maternal and paternal dietary patterns separately provide a comprehensive assessment and allow for an individualized approach to interventions by assessing their distinct associations with ART outcomes. Nevertheless, dietary patterns of partners in the same household can be influenced by each other, hindering the ability to fully distinguish their independent effects [109, 110]. To address this, we performed PCA separately for maternal and paternal data and accounted for maternal and paternal factors in the analyses by adjusting for potential confounders (e.g., age, and lifestyle factors) to better assess the independent effect of each parent. Analyzing couple-level dietary patterns and ART outcomes represents an interesting approach for future research, but is outside the scope of the study.

The findings of this study should be interpreted in light of the following limitations. First, embryo selection relied on morphological criteria only, which hindered us to determine whether associations between dietary patterns and embryo morphokinetics are translated into pregnancy and live birth outcomes. Studying the associations between embryo morphokinetics and other ART outcomes is outside the scope of the study. Second, the small sample size compromises the statistical power of the study and increases the likelihood of a type II error. Of note, a substantial number of eligible participants were excluded using the Goldberg method because of implausible dietary intake to ensure the reliability of dietary intake. The use of the Goldberg method might have also resulted in the exclusion of a substantial number of participants with overweight and obesity, which may underestimate the associations. Third, as this study included men and women attending the fertility clinic in a tertiary academic university hospital, despite a strong internal validity, the external validity through which the generalizability of the findings to other populations or to couples conceiving spontaneously may be limited. Nevertheless, the outpatient clinic provides IVF/ICSI services for the entire Rotterdam region and surrounding peripheral hospitals, which comprise a heterogeneous population (Table 1), thereby enhancing the representativeness of our study sample [111]. Also, annotations were performed for clinically relevant embryos i.e., embryos selected for transfer or cryopreservation, limiting the generalizability of the findings to discarded (non-viable) embryos. Discarded embryos were those that failed to reach the morula stage by day 4 (until 2019) or the full blastocyst stage by day 5 (after 2019). Of note, because of the developmental arrest, morphokinetic parameters of these embryos might be incomplete, and different from those with clinical potential. Further analyses of parental dietary patterns and the percentage of discarded embryos showed no significant associations (data not shown). Besides, there was low-to-moderate inter-observer agreement for the advanced embryo developmental stages (t6-t8), due to rapid and sometimes asynchronous cell divisions, as well as the increasing number of packed cells with overlapping boundaries, which negatively impacts data quality. Fourth, the four major dietary patterns explained only 33–34% of the total variance in diet. The remaining variance (~ 70%) was attributable to minor dietary patterns (each explaining < 6% of variance), indicating large variability in the diet, which was not examined in this study. Fifth, although we adjusted for parental covariates, missing dietary data from one partner (due to exclusion because of implausible dietary reporting or not completing the FFQ) may introduce potential confounding if this missingness is related to unmeasured factors that could influence both partners diets and the study outcomes. However, residual confounding is a limitation inherent to the observational nature of the study. Besides, after additional adjustment for subfertility factor and stimulation protocol, the associations between dietary patterns and embryo morphokinetics and yield remained consistent in direction and significance, except for paternal healthy pattern and t7 which lost significance (Supplementary Table 3). Sixth, due to the exploratory nature of the study, and consistent associations between parental dietary patterns and embryo morphokinetics, we did not correct for multiple testing, which increases the risk of a type I error. Therefore, the results should be interpreted with caution, and further studies are warranted to confirm these findings. Finally, sperm and oocyte quality were assessed using TMSC and the number of retrieved oocytes and MII oocytes; however, factors such as oxidative stress, mitochondrial function, DNA fragmentation might also be implicated. Therefore, we recommend that future studies investigate additional factors that reflect oocyte and sperm quality, particularly to establish causal relationships between diet and preimplantation embryo development.

Although PCA derives naturally occurring dietary patterns, these patterns might be challenging to translate into nutritional guidelines. Moreover, some patterns may include heterogeneous food combinations (e.g., highly loaded with healthy and unhealthy food), which can complicate the clinical interpretation. Nevertheless, our study found significant associations between a distinctly healthy (Healthy dietary pattern) and unhealthy pattern (Savory Snack and Alcohol-based pattern) with embryo morphokinetics, which can provide insights for dietary guidance. Another aspect that should be considered when using PCA-derived patterns is that the findings might not be reproducible into other populations. PCA is a data-driven approach that derives dietary patterns which are dependent on the dietary behaviours of the study sample, food items included, as well as methodological decisions of the investigator (e.g., number of factors retained, rotation method, addressing plausibility of intake). Nevertheless, the individual food components with high factor loadings in the pattern allow for (partial) reproducibility and comparability of patterns in different studies. For example, this can be done by investigating these specific food groups or items, or by applying a priori methods such as dietary indices.

## Conclusion

This exploratory study shows small but consistent positive associations between healthy maternal and pattern dietary patterns and faster preimplantation embryo development, as well as slower embryo development with maternal snack and alcohol-based dietary pattern. Due to the scarcity of data, investigating associations between dietary patterns and embryo morphokinetics, KIDscore D3 and D5 in large observational and intervention studies is required. Further investigation into preimplantation embryonic morphokinetics and ART outcomes, independent of embryonic morphological quality and KIDscore, is worthwhile. Despite the limited evidence, it is worth considering opportunities for interventions to promote a healthy diet among couples attending fertility clinic to improve preimplantation embryo outcomes.

## Supplementary Information

Below is the link to the electronic supplementary material.

Supplementary Material 1 (PDF 271 KB)

## Data Availability

The data that support the findings of this study are available from the corresponding author upon reasonable request.
