# Supplementary material for: Parental dietary patterns and assisted reproductive technology outcomes including embryo morphokinetics: rotterdam periconception cohort
Source: J Assist Reprod Genet. 2025 Oct 25;42(12):4347–60. doi: 10.1007/s10815-025-03688-y (PMC12705913; doi:10.1007/s10815-025-03688-y)
Supplement: Supplementary file 1 — Supplementary Material 1 (PDF 271 KB) [file 10815_2025_3688_MOESM1_ESM.pdf]

Parental dietary patterns and assisted reproductive technology outcomes including embryo morphokinetics: Rotterdam Periconception cohort

Journal of Assisted Reproduction and Genetics

Supplementary material

Supplementary information 1. Goldberg equation

$rEI:BMR_{rest}=PAL \times e^{(\pm 2 \times \frac{\frac{S}{100}}{\sqrt{n}})}$

Where rEI is the reported energy intake; BMR_{rest} is the resting basal metabolic rate; PAL is the physical activity level constant (PAL=1.55); S is the factor for variation in EI, BMR and PAL ($S=\sqrt{\frac{CV_{wEI}^2}{d}}$ x cv^2_{wB} x CV^2_{tP}); CV_{wEI} is the coefficient of within-subject variation in rEI; d is the days of dietary assessment (considered as habitual in this study); CV_{wB} is the coefficient of within-subject variation in repeated BMR measurements or the precision of estimated compared with measured BMR; and CV_{tP} is the total between-subject variation in PAL.

Supplementary information 2. Formulas for calculating the fertilization rate and embryo yield of IVF/ICSI treatments

$$\text{IVF: Fertilization rate} = \frac{\text{N of fertilized oocytes with two pronuclei}}{\text{N of retrieved oocytes}} \times 100$$

$$\text{ICSI: Fertilization rate} = \frac{\text{N of fertilized oocytes with two pronuclei}}{\text{N of MII oocytes}} \times 100$$

$$\text{Embryo yield} = \frac{\text{N usable embryos (transferred + frozen)}}{\text{N of fertilized oocytes with two pronuclei}} \times 100$$

Supplementary Table 1. Factor loadings of each food group of the four identified maternal and paternal dietary patterns using principle component analysis.

	Maternal dietary patterns				Paternal dietary patterns			
	**Healthy**	**Potato and Meat**	**Eggs, Legumes and Fruit and Vegetable Juices**	**Savory Snack and Alcohol**	**Healthy**	**Eggs and Meat**	**Potato-rich**	**Snack, Alcohol and Coffee**
Grains	-.366	-.176	-.084	-.240	-.025	-.385	.065	-.068
Savory snacks	-.057	.117	-.009	**.605**	-.030	-.006	-.017	**.658**
Alcoholic beverages	-.014	-.075	-.364	**.452**	-.147	.148	-.217	**.605**
butter	.102	-.007	-.387	.035	.092	-.088	.005	.226
cakes	.015	.171	-.363	.319	.032	-.251	.187	.166
coffee	-.059	-.276	-.332	.304	-.191	.212	.069	**.542**
Milk and dairy	.142	.247	-.156	-.060	-.041	-.084	.310	-.228
eggs	.228	.043	**.554**	.110	-.042	**.774**	-.120	-.088
Fish and shellfish	**.493**	-.215	.232	.276	.330	.014	.024	-.044
Fresh meat	.043	**.579**	.243	.111	-.094	**.696**	.223	-.167
Fruit	**.675**	-.291	.064	.021	**.586**	-.130	-.243	-.026
Fruit and vegetable juices	.004	-.103	**.506**	.103	-.423	.007	-.126	-.314
legumes	.328	-.209	**.569**	-.016	.220	.075	-.456	-.054

Margarines	-.257	.053	-.058	-.356	-.212	-.232	.127	-.133
potato	-.021	**.472**	-.063	-.395	.172	.039	**.723**	.141
Processed meat	-.148	**.576**	-.234	-.090	-.201	**.513**	.354	.075
sauces	-.240	.177	-.068	-.244	-.185	-.033	.357	**.558**
Soft drinks	-.422	.102	.365	.116	-.544	-.060	.102	-.316
soups	**.416**	.386	.012	.152	.248	-.101	-.024	**.407**
Soy and meat alternatives	.088	-.550	-.039	-.011	.211	-.109	-.477	-.010
Sugar and confectionary	-.571	-.041	.169	.075	-.179	-.297	.270	-.119
tea	.387	.075	-.055	.047	**.548**	-.117	-.053	-.285
Vegetable oils	.044	.065	-.196	-.604	.008	**.655**	.323	.092
vegetables	**.621**	-.363	.013	-.247	**.717**	.359	-.062	.028
Miscellaneous fat	-.185	.055	.268	.198	.160	.063	.365	-.129
Nuts and seeds	**.536**	-.070	.145	-.044	**.410**	-.121	-.107	-.042
Olive oil	.333	-.619	.114	-.001	.337	-.035	-.604	-.090
Variance (%)	10.493	8.606	7.171	6.670	9.130	8.982	8.366	7.849

Factor loadings of ≥0.4 are presented in bold.

Supplementary Table 2. Associations between maternal dietary patterns and oocyte quality (2a), and paternal dietary patterns and total motile sperm count (TMSC) (2b).

**Supplementary Table 2a**	**Healthy**	**Potato and meat**	**Eggs, legumes and fruit and vegetable juice**	**Savory snack and alcohol**
Number of Retrieved oocytes[a]				
β	-0.26	-0.11	-0.97	0.32
95%CI	-1.36, 0.84	-1.17, 0.96	-2.14, 0.20	-0.81, 1.44
P-value	0.639	0.843	0.105	0.580
Number of MII oocytes[a]				
β	-0.18	-0.36	-0.54	-0.26
95%CI	-1.67, 1.32	-1.64, 0.92	-2.33, 1.25	-1.65, 1.14
P-value	0.815	0.575	0.549	0.715
**Supplementary Table 2b**	**Healthy**	**Egg and meat**	**Potato- rich**	**Snack, alcohol and coffee**
TMSC[b]				
β	15.82	2.62	-14.06	4.99
95%CI	-0.22, 30.85	-6.51, 41.92	-31.03, 2.86	-6.71, 17.36
P-value	0.054	0.774	0.106	0.400

Abbreviations: β, Beta coefficient; CI, confidence interval; MII, metaphase II. N of women and men included in the analysis: 148 (retrieved oocytes), 88 (MII oocytes), and 126 (TMSC).

[a] Linear regression model adjusted for maternal age, BMI, geographic origin, daily calorie intake, smoking, alcohol consumption and dietary supplement use.

[b] Linear regression model with 10000 bootstrapping samples adjusted for paternal age, BMI, geographic origin, daily calorie intake, smoking, alcohol consumption and dietary supplement use.

Supplementary Table 3. Additional analyses of the associations between maternal (3a) and paternal (3b) dietary patterns, and embryo morphokinetics and yield after adjustment for additional covariates.

**Supplementary Table 3a**	**Healthy**	**Potato and meat**	**Eggs, legumes and fruit and vegetable juice**	**Savory snack and alcohol**
t6-tPNf				
β (95%CI)	0.27 (-0.52, 1.06)	-0.61 (-1.41, 0.18)	0.85 (-0.07, 1.77)	**0.83 (0.09, 1.56)**
p-value	0.496	0.125	0.069	**0.028**
t7-tPNf				
β (95%CI)	0.19 (-0.81, 1.19)	-0.14 (-1.16, 0.89)	0.23 (-0.95, 1.40)	**1.74 (0.85, 2.62)**
p-value	0.702	0.789	0.702	**0.0002**
t8-tPNf				
β (95%CI)	-0.12 (-1.44, 1.20)	-0.21 (-1.61, 1.18)	0.44 (-1.12, 2.00)	**1.76 (0.53, 2.99)**
p-value	0.852	0.761	0.577	**0.005**
S2				
β (95%CI)	**-0.68 (-1.23, -0.13)**	0.13 (-0.47, 0.74)	**-0.77 (-1.45, -0.09)**	0.12 (-0.45, 0.70)
p-value	**0.016**	0.661	**0.0277**	0.666
KID3				
OR (95%CI)	**0.75 (0.60, 0.93)**	0.82 (0.65, 1.05)	0.90 (0.67, 1.20)	1.23 (0.98, 1.55)
p-value	**0.010**	0.112	0.462	0.078
Embryo yield				
β, 95%CI [a]	1.35 (-4.83, 7.53)	-1.27 (-7.46, 4.92)	-4.27 (-11.17, 2.62)	**6.25 (0.34, 12.16)**
	0.665	0.684	0.221	**0.038**
**Supplementary Table 3b**	**Healthy**	**Egg and meat**	**Potato- rich**	**Snack, alcohol and coffee**
t2-tPNf				
β (95%CI)	-0.10 (-0.50, 0.29)	-0.14 (-0.51, 0.23)	**-0.45 (-0.79, -0.10)**	0.21 (-0.16, 6.23)
p-value	0.598	0.444	**0.012**	0.268
t7-tPNf				
β (95%CI)	-1.02 (-2.17, 0.13)	-0.55 (-1.63, 5.35)	-0.67 (-1.72, 0.37)	0.11 (-0.99, 1.21)
p-value	0.08	0.317	0.203	0.840
t8-tPNf				
β (95%CI)	**-1.99 (-3.30, -0.68)**	0.02 (-1.32, 1.36)	-0.76 (-2.03, 0.50)	0.007 (-1.32, 1.33)
p-value	**0.003**	0.977	0.232	0.992
S3				
β (95%CI)	**-1.70 (-2.78, -0.61)**	0.12 (-1.02, 1.26)	-0.18 (-1.24, 0.88)	-0.37 (-1.50, 0.76)
p-value	**0.002**	0.834	0.731	0.514

Abbreviations: β, Beta coefficient; CI, confidence interval; KID3, Known Implantation Data Day 3; OR, odds ratio; S2, second cell cycle synchrony; S3, third cell cycle synchrony; tPNf, timing to pronuclei fade; t2-t8, timing to form 2- to 8-cells division stages.

Model adjusted for maternal and paternal age, BMI, geographic origin, daily calorie intake, smoking, alcohol consumption, and dietary supplement use, conception mode, subfertility factor (male subfertility, endometriosis, ovulatory/cycle disorders), and stimulation protocol.
